# Association of HIV diversity and virologic outcomes in early antiretroviral treatment: HPTN 052

**DOI:** 10.1371/journal.pone.0177281

**Published:** 2017-05-08

**Authors:** Philip J. Palumbo, Ethan A. Wilson, Estelle Piwowar-Manning, Marybeth McCauley, Theresa Gamble, Newton Kumwenda, Joseph Makhema, Nagalingeswaran Kumarasamy, Suwat Chariyalertsak, James G. Hakim, Mina C. Hosseinipour, Marineide G. Melo, Sheela V. Godbole, Jose H. Pilotto, Beatriz Grinsztejn, Ravindre Panchia, Ying Q. Chen, Myron S. Cohen, Susan H. Eshleman, Jessica M. Fogel

**Affiliations:** 1 Dept. of Pathology, Johns Hopkins Univ. School of Medicine, Baltimore, Maryland, United States of America; 2 Vaccine and Infectious Disease Division, Fred Hutchinson Cancer Research Center, Seattle, Washington, United States of America; 3 Science Facilitation Department, FHI 360, Washington DC, United States of America; 4 Science Facilitation Department, FHI 360, Durham, North Carolina, United States of America; 5 College of Medicine-Johns Hopkins Project, Blantyre, Malawi; 6 Botswana-Harvard AIDS Institute Partnership, Gaborone, Botswana; 7 CART CRS, YRGCARE Medical Centre, VHS, Chennai, India; 8 Research Institute for Health Sciences, Chiang Mai University, Chiang Mai, Thailand; 9 Dept. of Medicine, Univ. of Zimbabwe, Harare, Zimbabwe; 10 Division of Infectious Diseases, Univ. of North Carolina at Chapel Hill, Chapel Hill, North Carolina, United States of America; 11 UNC Project-Malawi, Institute for Global Health and Infectious Diseases, Lilongwe, Malawi; 12 Hospital Nossa Senhora da Conceição, Serviço de Infectologia, Porto Alegre, Brazil; 13 National AIDS Research Institute (ICMR), Pune, India; 14 Hospital Geral de Nova Iguacu and Laboratorio de AIDS e Imunologia Molecular-IOC/Fiocruz, Rio de Janeiro, Brazil; 15 Instituto Nacional de Infectologia Evandro Chagas-INI-Fiocruz, Rio de Janeiro, Brazil; 16 Univ. of the Witwatersrand, Perinatal HIV Research Unit, Soweto HPTN CRS, Soweto, South Africa; 17 Vaccine and Infectious Disease Division, Fred Hutchinson Cancer Research Center, Seattle, Washington, United States of America; 18 Dept. of Medicine, Univ. of North Carolina at Chapel Hill, Chapel Hill, North Carolina, United States of America; Rush University, UNITED STATES

## Abstract

Higher HIV diversity has been associated with virologic outcomes in children on antiretroviral treatment (ART). We examined the association of HIV diversity with virologic outcomes in adults from the HPTN 052 trial who initiated ART at CD4 cell counts of 350–550 cells/mm^3^. A high resolution melting (HRM) assay was used to analyze baseline (pre-treatment) HIV diversity in six regions in the HIV genome (two in *gag*, one in *pol*, and three in *env*) from 95 participants who failed ART. We analyzed the association of HIV diversity in each genomic region with baseline (pre-treatment) factors and three clinical outcomes: time to virologic suppression after ART initiation, time to ART failure, and emergence of HIV drug resistance at ART failure. After correcting for multiple comparisons, we did not find any association of baseline HIV diversity with demographic, laboratory, or clinical characteristics. For the 18 analyses performed for clinical outcomes evaluated, there was only one significant association: higher baseline HIV diversity in one of the three HIV *env* regions was associated with longer time to ART failure (p = 0.008). The HRM diversity assay may be useful in future studies exploring the relationship between HIV diversity and clinical outcomes in individuals with HIV infection.

## Introduction

HIV genetic diversity generally increases with duration of infection [1, 2] and is influenced by viral factors and the host immune response [3, 4]. Antiretroviral treatment (ART) may decrease viral diversity [5]. Higher pre-treatment HIV diversity has been associated with high viral load [6], less effective control of viremia after strategic treatment interruption [7], and more rapid disease progression [8].

We developed a high resolution melting (HRM) assay for quantifying HIV diversity without sequencing [9, 10]. The HRM diversity assay measures the melting range of DNA amplicons generated from viral RNA and generates a single numeric HRM score that reflects the level of diversity in the genomic region analyzed [9]. Higher HRM scores are associated with higher viral diversity [10] and are highly correlated with diversity measures obtained using next-generation sequencing [10]. HRM scores were shown to be highly reproducible in a validation study that described the performance characteristics of the HRM diversity assay [11]. Furthermore, the following factors did not have a significant impact on results obtained with the HRM assay: HIV viral load, plasma sample volume, and number of HIV RNA copies used for DNA template preparation [11].

The HRM diversity assay has been used to quantify diversity in multiple regions in the HIV genome in large sample sets [1, 12–14]. Using this assay, we previously observed that higher pre-treatment *pol* diversity was associated with better treatment outcomes in children, including shorter time to viral suppression and longer time to ART failure [13]. The association between pre-treatment HIV diversity and treatment outcomes may vary in different patient populations and settings, reflecting viral factors (e.g., HIV subtype; viral replication mutation rates; pre-treatment HIV drug resistance), host factors (e.g., duration of infection; pre-treatment CD4 cell count and HIV viral load; serologic or cell-mediated immune responses to HIV infection), or clinical factors (e.g., treatment regimens; adherence to treatment). The association between pre-treatment HIV diversity and treatment outcomes is also likely to vary in different regions of the HIV genome, since the degree of viral diversity and the rate of viral diversification vary across the HIV genome, and since different regions of the HIV genome (e.g., *env*, *gag*, *pol*) are subjected to different selective pressures, both before and after treatment initiation [15]. The relationship between pre-treatment viral diversity and treatment outcomes are likely to be complex. For example, higher viral replication and/or mutation rates could be associated with higher pre-treatment diversity and could also favor emergence of resistant variants after treatment initiation, leading to worse treatment outcomes. Alternatively, high pre-treatment diversity could reflect a more robust immune response to HIV infection, which could enhance viral suppression after treatment initiation, leading to better treatment outcomes. In this report, we evaluated factors associated with pre-treatment HIV diversity and the relationship between pre-treatment HIV diversity and treatment outcomes in HIV-infected adults who initiated ART at higher CD4 cell counts.

## Methods

### Study cohort

Samples were obtained from the HIV Prevention Trials Network (HPTN) 052 trial, a Phase 3, randomized controlled trial, that enrolled HIV-1 serodiscordant couples at 13 sites in Africa, Asia, and the Americas (NCT00074581) [16, 17]. HIV-infected index participants had a CD4 cell count between 350–550 cells/mm^3^ at enrollment and were randomized to start ART immediately (early ART arm) or once their CD4 cell count fell below 250 cells/mm^3^ on two consecutive visits or upon development of an AIDS-defining condition (delayed ART arm). Participants reported that they were ART naïve prior to enrollment; prior short-term ARV use for prevention of mother-to-child transmission was permitted [16, 17]. In this study, we analyzed enrollment (baseline/pre-treatment) samples from index participants in the early ART arm who failed ART by May 2011; after May 2011, all index participants were offered ART at any CD4 cell count based on interim study findings.

### Laboratory methods

HIV viral load and CD4 cell count testing were performed at study sites. HIV genotyping was performed at four study sites (Pune and Chennai, India; Johannesburg, South Africa; Rio de Janeiro, Brazil) and at the HPTN Laboratory Center (Baltimore, MD); this testing was performed using the ViroSeq HIV-1 Genotyping System (Abbott Molecular, Des Plaines, IL). HIV subtypes were determined by phylogenetic methods, as previously described [18].

Plasma samples from index participants with baseline viral loads >400 copies/mm^3^ were analyzed with the HRM diversity assay, as previously described [1, 9]. In short, HIV diversity was quantified in six regions of the HIV genome: GAG1 (HXB2:1998–2097), GAG2 (HXB2:2068–2278), POL (HXB2:2373–2597), ENV1 (HXB2:7798–8036), ENV2 (HXB2:7950–8119), and ENV3 (HXB2:8016–8299). Region-specific amplification of template DNA was performed in the presence of a fluorescent, duplex-dependent, intercalating dye. The amplicons were melted using a LightScanner instrument (Model HR 96, BioFire Diagnostics Inc., Salt Lake City, UT) and the release of dye during melting was quantified. An automated software tool (DivMelt, version 1.0.2) was used to calculate the HRM score for each region in each sample; HRM scores are defined as the temperature range over which DNA melting occurred [10].

### Statistical analysis

Viral suppression was defined as occurring at the first of two study visits after ART initiation where the viral load was ≤400 copies/mL. ART failure was defined as occurring at the first of two study visits after 24 weeks on ART where the viral load was >1,000 copies/mL. Chi-square and Wilcoxon rank sum tests were used for categorical and continuous variables, respectively. Median regression analyses were used to evaluate associations between HRM scores and baseline demographic and clinical characteristics for each region. Cox proportional hazards regression was used to assess associations between each region and time to virologic suppression or time to ART failure; logistic regression was used to assess associations between each region and ARV resistance at failure. The Benjamini-Hochberg correction [19] was used to adjust for multiple comparisons, using a false discovery rate of 0.20. All statistical analyses were performed using SAS version 9.4.

### Ethical considerations

Institutional Review Boards/Ethics Committees at each participating institution approved the HPTN 052 trial (S1 Table). Written informed consent was obtained from all study participants.

## Results

### Samples used for analysis

In HPTN 052, 95 index participants in the early ART arm failed ART by May 2011. Baseline (pre-ART) HRM scores were obtained for six regions of the HIV genome (GAG1, GAG2, POL, ENV1, ENV2, and ENV3) for 86 (90.5%) of the 95 participants; nine participants were excluded from analysis (two had pre-ART viral loads ≤400 copies/mL; six had no baseline sample available; one sample failed amplification in the gp41 region, S2 Table). No significant differences in baseline demographic, laboratory, or clinical characteristics were observed between those who were vs. were not included in the study (S3 Table). HIV subtypes were determined for 85/86 samples. The majority of the samples were subtype C (n = 66). The HIV subtypes of the other 19 samples were: B (n = 9), F1 (n = 4), A1 (n = 3), A2 (n = 1), CRF1_AE (n = 1), and unique recombinant (n = 1).

### Factors associated with baseline HIV diversity

Associations between baseline HIV diversity and demographic, laboratory and clinical characteristics were evaluated using HRM scores generated for each region (Table 1).

**Table 1:** Association of baseline HIV diversity with baseline demographic, laboratory, and clinical characteristics*.

Variable	GAG1	GAG2	POL	ENV1	ENV2	ENV3
Sex	0.53	0.66	0.31	0.39	0.13	0.51
Age category	0.75	0.87	1.00	0.53	0.89	0.46
Education level	0.88	0.17	0.67	0.61	0.99	1.00
Geographical region	0.19	0.28	0.52	0.78	0.017	0.50
CD4 (per 100 CD4 increment)	1.00	0.58	0.39	0.15	0.38	0.30
VL (per unit log_10_ VL increment)	0.74	0.78	0.52	0.28	0.51	0.32
ARV resistance	0.19	0.84	0.39	0.07	1.00	0.049
Previous ARV for PMTCT	0.47	0.10	0.28	0.33	0.58	0.58

Abbreviations: VL: viral load; ARV: antiretroviral; PMTCT: prevention of mother-to-child transmission.

* Univariate quantile (median) regression analysis was used for all variables. None of the p-values was considered significant after adjusting for multiple comparisons using the Benjamini-Hochberg correction.

In univariate analysis, HRM scores for ENV2 were associated with geographical region (p = 0.017); this difference was not statistically significant when the data were corrected for multiple comparisons. The median (interquartile range [IQR]) ENV2 HRM scores were 5.4 (4.5, 6.0) for South America, 5.1 (4.4, 5.6) for Asia, and 4.4 (4.1, 5.4) for Africa (S1 Fig). ENV2 HRM scores were lower for subtype C than non-subtype C HIV (median [IQR]: 4.73 [4.15–5.54] vs. 5.21 [4.61–5.94]), but this difference was not statistically significant (p = 0.39). There was a trend of lower ENV3 diversity and baseline drug resistance (p = 0.049). However, this association was not statistically significant when the data were corrected for multiple comparisons.

### Association of baseline HIV diversity and treatment outcomes

Overall, 39.5% of the 86 participants were virally suppressed by 6 months, the median time to ART failure was 8.8 months (IQR: 6.0–11.9), and 33.7% had ARV drug resistance at ART failure. The associations of baseline diversity with time to viral suppression, time to ART failure, and ARV drug resistance at ART failure were evaluated using a dichotomous measure (> median HRM score vs. ≤ median HRM score for each region, Table 2).

**Table 2:** Associations of baseline HIV diversity and treatment outcomes*.

Region	Outcome	Hazard/Odds Ratio (95% CI)	p-value
GAG1	Time to viral suppression	1.20 (0.63, 2.30)	0.58
	Time to ART failure	0.97 (0.63, 1.50)	0.90
	Resistance at failure	0.53 (0.20, 1.37)	0.19
GAG2	Time to viral suppression	1.39 (0.73, 2.66)	0.32
	Time to ART failure	0.74 (0.48, 1.14)	0.17
	Resistance at failure	0.67 (0.27, 1.68)	0.40
POL	Time to viral suppression	1.22 (0.64, 2.33)	0.54
	Time to ART failure	0.74 (0.48, 1.15)	0.19
	Resistance at failure	0.62 (0.24, 1.58)	0.32
ENV1	Time to viral suppression	1.67 (0.87, 3.21)	0.12
	Time to ART failure	0.56 (0.36, 0.86)	**0.008**
	Resistance at failure	0.42 (0.16, 1.10)	0.08
ENV2	Time to viral suppression	0.86 (0.45, 1.66)	0.66
	Time to ART failure	1.25 (0.81, 1.95)	0.31
	Resistance at failure	0.62 (0.25, 1.57)	0.32
ENV3	Time to viral suppression	0.99 (0.52, 1.88)	0.97
	Time to ART failure	1.11 (0.72, 1.73)	0.63
	Resistance at failure	0.50 (0.20, 1.27)	0.15

Abbreviations: CI: confidence interval; ART: antiretroviral therapy.

* Hazard ratios for both time to viral suppression (censored at 12 months after ART initiation) and time to ART failure were estimated using Cox proportional hazards regression. Odds ratios for antiretroviral drug resistance at failure were estimated using logistic regression. In all analyses, viral regions were modelled using binary indicators based on median HRM score, with the reference as the group at or below the median HRM score. The Benjamini-Hochberg correction was used to adjust for multiple comparisons; a single p-value was statistically significant (shown in bold text).

Higher baseline ENV1 HRM scores were associated with longer time to ART failure (hazard ratio [HR]: 0.56, 95% confidence intervals [CI]: 0.36–0.89, p = 0.008); this association was still statistically significant when the data were corrected for multiple comparisons. The association of ENV1 diversity and time to ART failure was further supported by Kaplan-Meier analysis (S2 Fig). Diversity in GAG1, GAG2, POL, ENV2, and ENV3 was not associated with any of the three treatment outcomes. Other factors, such as baseline viral load, baseline CD4 cell count, and age were not associated with time to viral suppression or time to ART failure (data not shown). ENV1 HRM scores were similar for subtype C vs. non-subtype C HIV (median [IQR]: 4.65 [4.10–5.23]) vs. 4.50 [4.01–4.91]), but this difference was not statistically significant (P = 1.00).

## Discussion

In this study, we analyzed baseline HIV diversity among HIV-infected participants from the HPTN 052 trial who initiated early ART and subsequently failed treatment. We measured HIV diversity in two regions in *gag* (GAG1, GAG2), one region in *pol* (POL), and three regions in *env* (ENV1, ENV2, ENV3) using the HRM diversity assay. After correcting for multiple comparisons, we did not observe any association between baseline (pre-treatment) HIV diversity and any of the demographic, laboratory, or clinical characteristics evaluated. We also did not observe any associations between HIV diversity and time to viral suppression or ARV resistance at ART failure. A significant association was observed for higher baseline diversity in one of the three *env* regions (ENV1) and longer time to ART failure; diversity in the other five regions analyzed, including two other regions in *env*, was not associated with this outcome. The association between ENV1 and time to ART failure was not confounded by other factors, such as viral load, CD4 cell count, or age. In another study of participants in HPTN 052, those factors were associated with treatment outcomes [20]. Previous studies have examined the relationship between HIV diversity and viral load. One study found an association between higher *env* diversity and higher viral load [21]. Another study found an association between higher *gag* diversity and higher viral load, with no association between *env* diversity and viral load [15]. In our previous study of HIV-infected children, we found no association between viral load and diversity in *gag* or *pol* and with a borderline association in *env* [13].

Higher levels of *env* diversity prior to ART initiation might reflect a higher baseline viral replication rate and/or a higher viral mutation rate; either of these factors could make it more difficult to suppress viral replication with ART. The association of higher pre-treatment ENV1 diversity and longer time to ART failure that we observed in this study differs from our findings in a pediatric cohort. In that study, higher pre-treatment ENV2 diversity was associated with a worse treatment outcome (shorter time to ART failure) [13]; ENV1 diversity was not associated with any treatment outcomes in that study [13]. In another pediatric study, we found that higher pre-treatment ENV1 and ENV2 diversity was associated with lack of viral suppression, another bad outcome [14]. It is difficult to compare results from HPTN 052 and these pediatric cohorts, since the treatment regimens were different and many other factors could have impacted treatment outcomes.

The different associations that we observed for HIV diversity in the ENV1 region and other regions analyzed (ENV2, ENV3, GAG1, GAG2, POL) could reflect differences in the mutation rates in these regions of the HIV genome. Alternatively, the different associations could reflect differences at the protein level. For example, anti-HIV antibodies and cytotoxic T lymphocytes (CTLs) target different regions of the HIV genome; this could impact diversification in these regions if selective pressures varied from one region to another. Different domains of HIV proteins encoded by each HRM region may also vary in the degree of structural constraint; this could hinder or facilitate diversification in the respective coding regions of the HIV genome. In this study, higher diversity in the ENV1 region was associated with a longer time to virologic failure. ENV1 encodes the heptad repeat 1 (HR1) region of gp41 and regions to either side of that domain. ENV2 encodes the immunodominant region (IDR) cluster 1 of gp41 and portions of HR1 and heptad repeat 2 (HR2). ENV3 encodes HR2 and regions to either side of that domain [22]. It is not clear why diversity in these regions would have different associations with clinical outcomes in this study and previous reports. It is also possible that the statistically significant association observed in this study did not reflect a biologic association, but was due to chance.

This study was also limited to participants in the early ART arm of HPTN 052, who started and failed ART before interim study results were released (before May 2011). In this group, participants initiated ART at higher CD4 cell counts; they did not know if ART would prevent HIV transmission to their partners, or if there were any health benefits or risks associated with early ART. This may have impacted adherence to treatment. Another limitation is that treatment adherence was not evaluated in this study. Of note, we did not find an association of HIV diversity and treatment outcomes when we examined different regions of *env*. Variation in genetic diversity in different sub-regions of HIV *env* may reflect variations in targeting of antibodies or cytotoxic T lymphocyte (CTL) responses to different epitopes on envelope proteins; however, serologic data was not available in this study. Variations in the mutation rate in different regions of the *env* gene, or other factors may also impact *env* diversity. These results highlight the complexity of the relationship between HIV diversity and treatment outcomes.

In this report, we found an association of ENV2 diversity and geographical region, with the highest diversity in South America and the lowest diversity in Africa. This association did not appear to reflect differences in HIV subtype, since ENV2 HRM scores were similar in participants infected with subtype C vs. non-subtype C HIV. This regional difference in ENV2 diversity was not observed when the data were corrected for multiple comparisons. However, because some of the variables included in this analysis may not be independent, the correction may mask a true association.

*Env* diversity has also been associated with differences in disease progression, which was not examined in this report. Those studies have produced conflicting results. Studies using sequencing methods to quantify diversity have observed associations between higher *env* diversity and either more rapid disease progression [7, 8, 23, 24] or slower disease progression [25–27]. The different methods for testing and different HIV genomic regions analyzed in those studies make it difficult to compare results across the studies.

The HRM diversity assay is a high-throughput assay that can be used to analyze multiple genomic regions in large sample sets. For example, this study included six diversity measures in 86 samples (516 diversity measures). This approach for quantifying HIV diversity may be useful in future studies evaluating the complex relationship between HIV diversity, ART outcomes, and other clinical outcomes in HIV infection.

## Supporting information

S1 Fig Distribution of baseline ENV2 HRM scores by geographical region. The box plot shows the distribution of ENV2 HRM scores by geographical region (South America [n = 17], Asia [n = 26], and Africa [n = 43]). Median (interquartile range [IQR]) ENV2 HRM scores were 5.4 (4.5, 6.0) for South America, 5.1 (4.4, 5.6) for Asia, and 4.4 (4.1, 5.4) for Africa. Abbreviations: HRM: high resolution melting.
(PDF)

S2 Fig Kaplan-Meier plot showing the probability of ART failure as a function of time for participants with ENV1 HRM scores above vs. below the median. Kaplan-Meier plot showing the association of higher ENV1 HRM score (>median) with longer time to antiretroviral (ART) failure. The numbers below the graph indicate the number (N) of participants failing ART per time point.
(PDF)

S1 Table Affiliated IRBs/ECs and regulatory bodies by site. (PDF)

S2 Table High resolution melting (HRM) scores, geographical region, and HIV subtype of HIV-infected adults analyzed using the HRM diversity assay in HPTN 052. (PDF)

S3 Table Enrollment (baseline) characteristics of the study cohort (N = 95)*. (PDF)
