# Supplementary material for: Association of HIV diversity and virologic outcomes in early antiretroviral treatment: HPTN 052
Source: PLoS One. 2017 May 8;12(5):e0177281. doi: 10.1371/journal.pone.0177281 (PMC5421787; doi:10.1371/journal.pone.0177281)
Supplement: S1 Fig — The box plot shows the distribution of ENV2 HRM scores by geographical region (South America [n = 17], Asia [n = 26], and Africa [n = 43]). Median (interquartile range [IQR]) ENV2 HRM scores were 5.4 (4.5, 6.0) for South America, 5.1 (4.4, 5.6) for Asia, and 4.4 (4.1, 5.4) for Africa. Abbreviations: HRM: high resolution melting. (PDF) [file pone.0177281.s001.pdf]

S1 Fig. Distribution of baseline ENV2 HRM scores by geographical region.

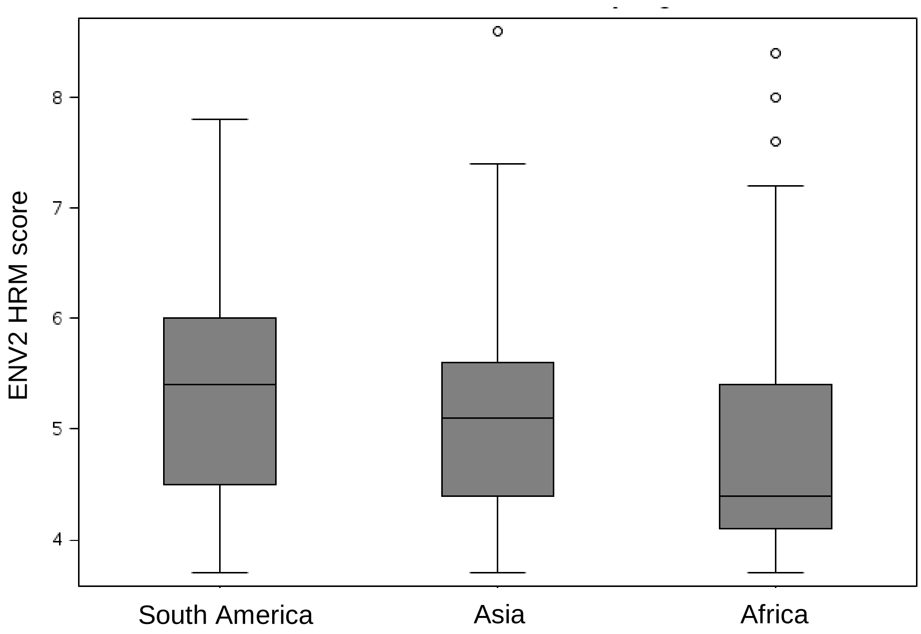

Legend for S1 Fig

The box plot shows the distribution of ENV2 HRM scores by geographical region (South America [n=17], Asia [n=26], and Africa [n=43]). Median (interquartile range [IQR]) ENV2 HRM scores were 5.4 (4.5, 6.0) for South America, 5.1 (4.4, 5.6) for Asia, and 4.4 (4.1, 5.4) for Africa.

<u>Abbreviations</u>: HRM: high resolution melting.
