# Supplementary material for: Association of HIV diversity and virologic outcomes in early antiretroviral treatment: HPTN 052
Source: PLoS One. 2017 May 8;12(5):e0177281. doi: 10.1371/journal.pone.0177281 (PMC5421787; doi:10.1371/journal.pone.0177281)
Supplement: S2 Fig — Kaplan-Meier plot showing the association of higher ENV1 HRM score (>median) with longer time to antiretroviral (ART) failure. The numbers below the graph indicate the number (N) of participants failing ART per time point. (PDF) [file pone.0177281.s002.pdf]

S2 Fig. Kaplan-Meier plot showing the probability of ART failure as a function of time for participants with ENV1 HRM scores above vs. below the median.

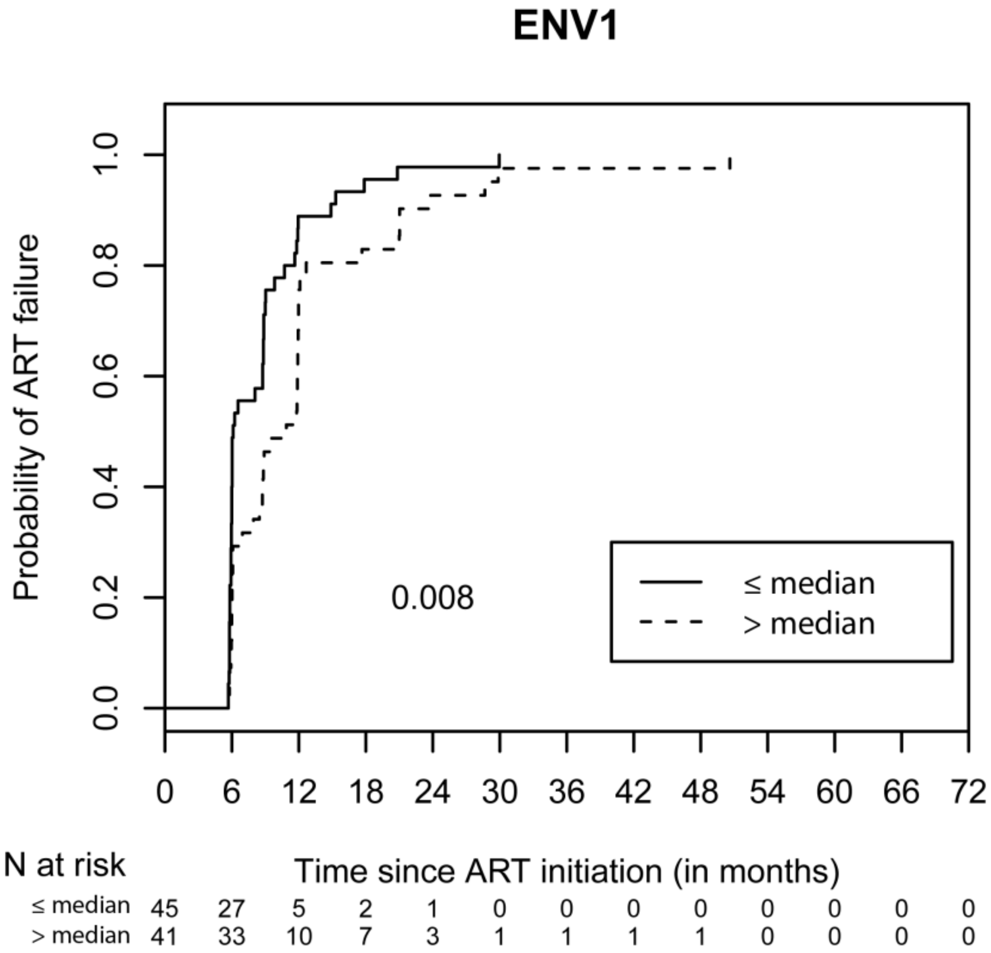

Legend for S2 Fig

Kaplan-Meier plot showing the association of higher ENV1 HRM score (>median) with longer time to antiretroviral (ART) failure. The numbers below the graph indicate the number (N) of participants failing ART per time point.
