# Supplementary material for: Association of HIV diversity and virologic outcomes in early antiretroviral treatment: HPTN 052
Source: PLoS One. 2017 May 8;12(5):e0177281. doi: 10.1371/journal.pone.0177281 (PMC5421787; doi:10.1371/journal.pone.0177281)
Supplement: S1 Table — (PDF) [file pone.0177281.s003.pdf]

S1 Table. Affiliated IRBs/ECs and Regulatory Bodies by Site.

The table below outlines all associated Institutional Review Boards (IRBs)/Ethics Committees for each clinical research site. In some cases, a local site partnered with an institution in the United States (US) to conduct HPTN 052. In those cases, a US IRB also had a responsibility to review and approve the research. The table also includes non-US regulatory bodies that reviewed the study (for example local Ministries of Health, Poisons Boards, etc.).

Site	Affiliated IRBs/ECs and Regulatory Bodies
Porto Alegre, Brazil	• UCLA Office for Protection of Research Subjects: Medical Institutional Review Board<br>• Brazil Ministério da Saúde: CONEP: Comissão Nacional de Ética em Pesquisa<br>• Gerencia de Ensino e Pesuisa: Comitê de Ética em Pesquisa do Grupo Hospitalar Conceição-GHC
Rio de Janeiro, Brazil	• UCLA Office for Protection of Research Subjects: Medical Institutional Review Board<br>• Instituto de Pesquisa Clínica Evandro Chagas: Comitê de Ética em Pesquisa<br>• Brazil Ministério da Saúde: CONEP: Comissão Nacional de Ética em Pesquisa<br>• Grupo Hospitalar Conceição-GHC: Comitê de Ética em Pesquisa
Boston, MA, USA	• Fenway Community Health Center: Fenway Community Health Center Institutional Review Board
Chennai, India	• Fenway Community Health Center: Fenway Community Health Center Institutional Review Board<br>• YRG CARE Institutional Review Board<br>• University of California, San Diego: Human Research Protections Program<br>• Health Ministry Screening Committee (India)
Pune, India	• National AIDS Research Institute: National AIDS Research Institute (NARI) Ethics Committee<br>• Johns Hopkins University School of Medicine: Johns Hopkins Medicine Institutional Review Board<br>• Health Ministry Screening Committee (India)
Chiang Mai, Thailand	• Johns Hopkins Bloomberg School of Public Health Institutional Review Boards<br>• Human Experimentation Committee, Research Institute for Health Sciences, Chiang Mai University<br>• Research Ethics Committee, Faculty of Medicine, Chiang Mai University<br>• Ethical Review Committee for Research in Human Subjects Ministry of Public Health, Thailand
Kisumu, Kenya	• Kenya Medical Research Institute: KEMRI National Ethical Review Committee<br>• CDC Atlanta: CDC National Center for HIV/AIDS, Viral Hepatitis, STDs and TB Prevention IRB<br>• Pharmacy and Poisons Board (Kenya)
Harare, Zimbabwe	• University of California at San Francisco: Committee on Human Research, Office of Research Administration<br>• Medical Research Council of Zimbabwe: Medical Research Council of Zimbabwe (MRCZ) Institutional Review Board
Blantyre, Malawi	• University of Malawi College of Medicine: College of Medicine Research & Ethics Committee (COMREC)<br>• Johns Hopkins University Bloomberg School of Public Health: Institutional Review Board
Lilongwe, Malawi	• Malawi Ministry of Health & Population: National Health Sciences Research Committee<br>• University of North Carolina School of Medicine: Committee on the Protection of the Rights of Human Subjects
Gaborone, Botswana	• Botswana Ministry of Health: Health Research and Development Committee<br>• Harvard School of Public Health: Human Subjects Committee
Johannesburg, South Africa	• University of Witwatersrand: Human Research Ethics Committee: Medical<br>• Medicines Control Council (South Africa)
Soweto, South Africa	• University of Witwatersrand: Human Research Ethics Committee: Medical<br>• Medicines Control Council (South Africa)
HPTN Laboratory Center	• Johns Hopkins University School of Medicine eIRB2
