# Supplementary material for: Association of HIV diversity and virologic outcomes in early antiretroviral treatment: HPTN 052
Source: PLoS One. 2017 May 8;12(5):e0177281. doi: 10.1371/journal.pone.0177281 (PMC5421787; doi:10.1371/journal.pone.0177281)
Supplement: S2 Table — (PDF) [file pone.0177281.s004.pdf]

S2 Table. High resolution melting (HRM) scores, HIV subtype, and geographical region of HIV-infected adults analyzed using the HRM diversity assay in HPTN 052.

#	HIV Subtype	Region	Resistance (yes/no)	Days to failure	HRM gag1	HRM gag2	HRM pol	HRM env1	HRM env2	HRM env3
1	C	Africa	no	361	6.0	5.5	3.5	4.4	4.2	7.9
2	A1	Africa	n/a	269	9.1	4.9	5.8	5.7	5.2	5.6
3	A1	Africa	no	267	4.7	7.0	3.6	4.0	4.6	5.1
4	A2	Africa	no	359	6.1	11.5	5.8	4.8	5.2	8.5
5	A1	Africa	no	176	4.9	5.0	4.0	4.0	4.2	6.1
6	C	Africa	no	210	5.4	5.7	5.5	4.7	4.2	4.5
7	C	Africa	no	357	4.5	4.8	4.3	6.1	4.9	4.5
8	C	Africa	no	181	5.6	6.7	5.8	5.3	3.8	6.4
9	C	Africa	no	180	5.1	4.8	6.1	5.8	4.1	3.6
10	C	Africa	no	182	6.4	6.5	6.0	4.3	5.1	5.6
11	C	Africa	no	185	4.5	5.3	7.3	4.0	5.7	5.7
12	C	Asia	yes	264	5.3	5.6	4.9	4.7	6.1	4.7
13	C	Asia	yes	636	9.5	3.9	7.2	5.7	4.5	5.1
14	C	Asia	yes	357	10.1	11.6	6.2	4.1	5.1	8.4
15	C	Asia	no	362	4.4	6.5	3.1	5.5	5.4	5.7
16	C	Asia	yes	297	6.4	9.6	4.7	4.1	5.6	7.0
17	n/a	Asia	n/a	720	6.6	4.8	6.1	4.8	4.2	5.3
18	C	Asia	no	181	6.3	6.0	5.8	5.0	3.9	4.4
19	C	Asia	yes	906	5.9	8.8	5.2	4.3	4.4	5.2
20	C	Asia	no	181	8.0	6.9	5.8	7.7	5.4	6.0
21	C	Asia	no	176	6.1	10.3	4.8	4.4	6.3	7.1
22	C	Asia	no	175	6.9	6.1	5.5	4.1	5.6	5.2
23	C	Asia	no	175	5.9	7.0	5.9	3.9	4.9	5.5
24	C	Asia	no	268	6.2	4.2	4.8	4.1	4.9	5.1
25	C	Asia	no	267	5.8	6.8	4.7	6.8	6.7	4.7
26	C	Asia	no	288	5.9	10.3	4.3	5.2	3.7	5.9
27	C	Asia	no	366	5.5	7.3	3.1	5.2	7.4	4.6
28	C	Asia	no	181	7.6	8.7	4.9	4.3	5.1	6.7
29	C	Asia	no	360	7.7	7.9	4.4	4.9	8.6	9.7
30	C	Asia	yes	184	5.0	5.7	4.8	4.7	5.0	6.1
31	C	Asia	no	182	6.4	6.0	5.7	4.1	5.1	5.9
32	C	Asia	no	867	4.4	11.0	6.0	5.2	4.2	6.4
33	C	Asia	yes	329	5.0	6.1	4.0	5.5	5.4	6.4
34	C	Asia	no	540	6.9	4.0	5.9	4.0	4.4	5.5
35	C	Asia	no	267	4.7	9.9	4.8	4.3	3.7	5.4
36	C	Asia	no	175	8.3	5.9	6.5	7.8	5.6	4.9
37	CRF1_AE	Asia	no	180	4.3	5.8	4.9	4.1	3.7	5.6
38	C	South America	no	362	5.5	4.7	5.4	6.5	3.7	7.6
39	C	South America	yes	182	5.1	9.4	5.5	5.3	4.3	5.3
40	C	South America	no	182	8.5	3.3	3.6	4.3	3.8	6.4
41	B	South America	yes	268	6.1	5.7	5.3	4.2	5.4	4.7

42	F1	South America	yes	189	6.1	5.3	6.4	4.4	4.6	8.7
43	F1	South America	no	175	4.5	5.0	3.8	3.7	4.7	4.6
44	B	South America	no	175	6.6	5.0	4.2	3.8	7.6	5.6
45	B	South America	no	265	6.0	5.2	4.3	3.6	5.8	5.6
46	F1	South America	no	179	5.2	5.2	5.4	4.6	4.5	3.0
47	B	South America	no	184	9.6	5.6	5.6	5.2	6.5	6.5
48	B	South America	n/a	534	5.9	10.1	4.9	4.9	4.4	7.4
49	URF	South America	no	352	4.5	6.5	4.2	4.1	5.0	6.1
50	F1	South America	no	383	6.1	7.9	6.0	5.0	5.8	5.4
51	B	South America	yes	174	5.4	7.3	4.3	4.6	7.6	5.5
52	B	South America	no	360	5.7	7.9	5.0	4.9	6.0	8.8
53	B	South America	no	362	5.7	7.6	5.3	5.7	7.8	5.8
54	B	South America	yes	181	4.9	5.4	4.9	4.5	5.8	4.1
55	C	Africa	yes	360	5.1	4.4	4.0	3.6	4.1	4.5
56	C	Africa	no	903	4.8	4.8	4.6	5.2	4.0	5.4
57	C	Africa	yes	630	5.1	6.6	4.4	4.5	4.3	5.5
58	C	Africa	yes	176	4.9	5.2	4.2	4.6	4.3	5.0
59	C	Africa	no	198	4.7	4.2	3.7	4.5	5.8	4.7
60	C	Africa	yes	179	5.0	5.4	3.3	3.7	3.9	4.6
61	C	Africa	no	266	8.0	10.0	6.3	4.8	8.0	9.0
62	C	Africa	no	635	4.4	9.9	7.3	6.0	4.0	5.7
63	C	Africa	no	256	4.9	5.6	5.3	5.0	5.7	4.2
64	C	Africa	yes	273	5.5	6.6	3.9	4.2	4.4	4.7
65	C	Africa	no	182	6.0	5.7	5.5	3.8	4.3	5.2
66	C	Africa	no	358	6.7	6.7	6.6	5.2	5.4	5.8
67	C	Africa	yes	181	4.5	4.5	4.1	3.6	3.9	3.9
68	C	Africa	no	450	5.9	6.6	3.8	4.4	5.3	6.1
69	C	Africa	no	182	7.7	7.2	4.3	5.4	4.3	8.1
70	C	Africa	yes	265	4.5	6.1	4.7	4.0	3.8	4.6
71	C	Africa	no	359	5.9	5.4	6.7	6.4	7.2	5.3
72	C	Africa	yes	180	4.4	4.8	4.6	3.8	4.0	4.1
73	C	Africa	yes	179	5.0	5.4	5.4	5.3	4.9	5.0
74	C	Africa	yes	272	6.3	6.1	4.9	4.3	8.4	4.5
75	C	Africa	yes	240	8.3	7.1	5.5	5.7	4.0	5.9
76	C	Africa	no	1531	6.1	6.2	6.0	5.0	7.6	4.0
77	C	Africa	no	172	4.5	5.5	4.9	4.3	5.7	4.4
78	C	Africa	yes	244	6.0	4.8	5.2	4.1	4.4	6.3
79	C	Africa	yes	176	6.3	5.2	4.9	4.8	7.2	6.2
80	C	Africa	no	361	6.8	4.9	3.7	4.9	4.3	5.2
81	C	Africa	yes	324	4.8	4.6	4.5	3.8	4.3	8.3
82	C	Africa	no	463	5.9	4.0	3.7	3.4	4.9	7.5
83	C	Africa	no	179	6.4	4.8	3.8	3.9	3.7	4.2
84	C	Africa	yes	172	4.7	4.3	4.0	4.3	3.9	4.3
85	C	Africa	no	636	6.1	4.4	4.2	4.9	4.6	5.2
86	C	Africa	no	172	4.6	8.7	4.3	4.8	5.5	5.9

Note: For access to additional data, please contact the corresponding author as these data are not yet publically available. CRF: circulating recombinant form; URF: unique recombinant form.
