# Supplementary material for: Association of HIV diversity and virologic outcomes in early antiretroviral treatment: HPTN 052
Source: PLoS One. 2017 May 8;12(5):e0177281. doi: 10.1371/journal.pone.0177281 (PMC5421787; doi:10.1371/journal.pone.0177281)
Supplement: S3 Table — (PDF) [file pone.0177281.s005.pdf]

S3 Table. Enrollment (baseline) characteristics of the study cohort (N=95).*

Variables	Included N=86	Excluded N=9	p-value
Sex			0.41
Male	45 (52.3%)	6 (66.7%)	
Female	41 (47.7%)	3 (33.3%)	
Age			0.68
<25 years	12 (14.0%)	2 (22.2%)	
25-39 years	56 (65.1%)	6 (66.7%)	
≥40 years	18 (20.9%)	1 (11.1%)	
Education			0.52
None	16 (18.6%)	2 (22.2%)	
Primary or secondary schooling	67 (77.9%)	6 (66.7%)	
Post-secondary schooling	3 (3.5%)	1 (11.1%)	
Geographical region			0.18
South America	17 (19.8%)	0 (0.0%)	
Asia	26 (30.2%)	5 (55.6%)	
Africa	43 (50.0%)	4 (44.4%)	
CD4 (per 100 CD4 cells/mm^3 increments)			0.94
Median (IQR)	462 (371, 535)	445 (413, 528)	
VL (per unit log_{10} increment)			0.07
Median (IQR)	4.5 (4.0, 4.9)	3.7 (2.6, 4.4)	
Antiretroviral drug resistance at baseline			0.42
Missing	4 (4.7%)	2 (22.2%)	
No	75 (87.2%)	7 (77.8%)	
Yes	7 (8.1%)	0 (0.0%)	
PMTCT			0.77
No	26 (74.3%)	2 (66.7%)	
Yes	9 (25.7%)	1 (33.3%)	
Number of sex partners			0.51
1	82 (95.3%)	9 (100.0%)	
>1	4 (4.7%)	0 (0.0%)	

Footnotes for S3 Table

Abbreviations: IQR: interquartile range; VL: viral load; PMTCT: prevention of mother-to-child transmission.

*Chi-square test was used for categorical variables and Wilcoxon rank sum test was used for continuous variables.
